# Assessment of Inflammation in 3D Reconstructed Human Skin Exposed to Combined Exposure to Ultraviolet and Wi-Fi Radiation

**DOI:** 10.3390/ijms24032853

**Published:** 2023-02-02

**Authors:** Zsófia Szilágyi, Zsuzsanna Németh, József Bakos, Györgyi Kubinyi, Péter Pál Necz, Erika Szabó, György Thuróczy, Rosanna Pinto, Brahim Selmaoui

**Affiliations:** 1 Non-Ionizing Radiation Unit, National Public Health Center, H-1221 Budapest, Hungary; 2 Division of Health Protection Technologies, Italian National Agency for New Technologies, Energy and Sustainable Economic Development (ENEA), Santa Maria di Galeria, 00123 Rome, Italy; 3 Department of Experimental Toxicology, National Institute of Industrial Environment and Risks (INERIS), 60550 Verneuil en Halatte, France; 4 PériTox Laboratory, UMR-I 01 INERIS, Picardie Jules Verne University, 80025 Amiens, France

**Keywords:** Wi-Fi, radiofrequency, UV, interleukin, skin, adaptive response, inflammation

## Abstract

In the human environment, the increasing exposure to radiofrequency (RF) radiation, especially that emitted by wireless devices, could be absorbed in the body. Recently, mobile and emerging wireless technologies (UMTS, DECT, LTE, and Wi-Fi) have been using higher frequencies than 2G GSM systems (900/1800 MHz), which means that most of the circulating RF currents are absorbed into the skin and the superficial soft tissue. The harmful genotoxic, cytotoxic, and mutagenic effects of solar ultraviolet (UV) radiation on the skin are well-known. This study aimed at investigating whether 2422 MHz (Wi-Fi) RF exposure combined with UV radiation in different sequences has any effect on the inflammation process in the skin. In vitro experiments examined the inflammation process by cytokines (IL-1α, IL-6, IL-8) and MMP-1 enzyme secretion in a 3D full-thickness human skin model. In the first study, UV exposure was immediately followed by RF exposure to measure the potential additive effects, while in the second study, the possible protective phenomenon (i.e., adaptive response) was investigated when adaptive RF exposure was challenged by UV radiation. Our results suggest that 2422 MHz Wi-Fi exposure slightly, not significantly increased cytokine concentrations of the prior UV exposure. We could not detect the adaptive response phenomenon.

## 1. Introduction

As part of the nonionizing electromagnetic spectrum, the radiofrequency (RF) electromagnetic field (EMF) frequency ranges from 30 kHz to 300 GHz. RFs emitted by different devices are generally used in telecommunications, industry, medicine, or by the military. The adoption of RF EMFs in wireless communication, such as Wi-Fi or Bluetooth, occurred in the last few decades. The exponential increase in RF exposure raises questions about their possible biological and health effects. Therefore, there is an ongoing concern in the public regarding the potential for adverse human health effects from such exposure, as taking steps into modern technologies, i.e., 5G and 6G, will result in higher RFs becoming omnipresent in modern society. As frequency increases, the penetration depth into the human tissue decreases, which means that most of the RF power (specific absorption rate, SAR (W/kg)) is absorbed in the skin of the body [1]. Safety standards, which limit the exposure of the human body in residential and work fields, are based on the well-described thermal effect [2]. Nonthermal effects occur when the intensity of RF radiation is adequately low so that the amount of energy involved would not significantly increase the temperature of a cell, tissue, or organism, but may induce some physical or biochemical changes. Regarding the international guidelines, thermal effects may occur when there is a more than 1 °C temperature increase in the human body. Adverse nonthermal health effects have not been established so far. Above 4 W/kg SAR of exposure, whole-body exposure results in a body core temperature increase > 1 °C, which is potentially harmful. Without the thermoregulation process (i.e., in vitro systems), above 1 W/kg SAR, the temperature may increase depending on the exposure conditions.

Nevertheless, to the best of our knowledge, the potential adverse health effect of RF magnetic fields is caused by thermal effects. A lot of studies observed this phenomenon [2]. Several reports then looked at the nonthermal effects of exposure to RFs on health, such as cancer, physiological functions [3,4,5], endocrine system [6], fertility, genotoxicity [7,8], sleep [9,10], etc. Most of the data did not find any effect, and few are inconclusive or found a significant effect. The effects of RF radiation depend on many factors (frequency, dose rate, waveform, modulation, exposure time, temperature, tissue or cell type, endpoint, and exposure condition) [11]. Many in vitro studies focus on genotoxic effects, such as single-strand breaks (SSBs), double-strand breaks (DSBs), genome structural aberrations, and reactive oxygen species (ROS), which are crucial in carcinogenesis. Most of the studies used single cell gel electrophoresis (comet assay) or identification of phosphorylated H2AX histone (γH2AX), which show SSBs and DSBs, and reported no DNA damage [12,13]. In the light of ‘‘weak mechanistic” evidence and “limited evidence” in humans and experimental animals, the working group of the International Agency for Research on Cancer (IARC) classified RF-EMF as “possibly carcinogenic to humans” (Group 2B) in 2011 [14].

Wi-Fi devices contain low-powered RF transceivers that support wireless local area networks (WLANs). Its most common usage is to provide the Internet to computers or mobile devices, as well as household devices (e.g., microwaves), which are present in homes, offices, and other environments. It is well-known that smartphones connect immediately to the available Wi-Fi access points. Therefore, public exposure to RFs emitted from smartphones at frequency bands used by Wi-Fi systems increases continuously. Although the levels of RF exposure from WLAN devices are far below the international limits—the exposure limit for the whole body is 0.08 W/kg, for the head, it is 2 W/kg, and 4 W/kg for the limbs [2,15]—it is important to research the potential health risk below and above these limits as well. In an in vitro study, Regalbuto et al. reported that 2450 MHz does not induce genotoxic and cytotoxic effects on fibroblasts at a SAR of 0.7 W/kg [16]. Schuermann et al. suggested that the possible carcinogenicity of modulated electromagnetic fields (i.e., GSM, UMTS Wi-Fi, RFID) cannot be explained by an effect on genome integrity through direct DNA damage; however, nongenotoxic, indirect effects that may promote tumorigenesis cannot be ruled out [17].

Another important question is whether RF radiation (classified by the IARC as Group 2B) in combination with other physical agents has any effect on the human body. Solar radiation is the main natural source of human exposure to ultraviolet (UV) radiation. Around 95% of UV radiation that reaches the Earth’s surface is UV-A (320–400 nm). Most of the solar UV-B (280–320 nm) and the whole range of UV-C (180–280 nm) are blocked by the stratospheric ozone [18]. UV radiation causes many adverse biological effects including photoaging and skin cancer. Based on these effects, UV radiation has been classified as a human carcinogen (Group 1) by the IARC in 2012 [19]. The response of the human body to UV exposure may be inflammation and photoaging of the skin [20,21]. The most common photoproducts of UV radiation formed in the skin are cyclobutane pyrimidine dimers (CPDs) and pyrimidine–pyrimidone (6–4) photoproducts (PPs) [22,23,24]. Many studies reported an increased level of CPDs and 6–4 PPs [25,26], and some studies noted a highly increased level of ROS [27]. In addition, UV radiation also results in DNA strand breaks and DNA crosslinks [22,28]. UV radiation may trigger cutaneous inflammatory responses by directly inducing epidermal keratinocytes to elaborate specific cytokines such as interleukin (IL-1) and IL-6 [29]. The viable and more basally located keratinocytes respond by releasing interleukin 1 alpha (IL-1α), initiating an acute inflammatory reaction. The proinflammatory cytokine IL-1α is considered a key inducer of the skin inflammatory cascade. After a short period, IL-1α diffuses to the adjacent dermis, and fibroblasts secrete secondary cytokines, such as IL-6 and IL-8. Moreover, UV radiation causes DNA damage, protein oxidation and induces matrix metalloproteinases (MMPs) [30]. UV-induced matrix metalloproteinase-1 (MMP-1) is a crucial biomarker of photoaging [31,32,33].

Studies of inflammation on the skin caused by EMF are very scarce. Kubat et al. found changes in the relative amount of (m)RNAs encoding the enzymes involved in heme catabolism and removal of reactive oxygen species [34]. In another study, Kim et al. showed that exposure to 1760 MHz RF-EMF significantly induces ROS generation, leading to MMP activation and FoxO3a and ERK1/2 phosphorylation [35].

Adaptive response (AR) describes the phenomenon when cells which were pre-exposed to extremely low and nontoxic doses of a genotoxic agent (as an adaptive dose, AD) became resistant to the damage induced by subsequent exposure to a higher and toxic dose of the same, similar (in action), or another genotoxic agent (as a challenge dose, CD). Adaptive response is a well-known phenomenon in the research field of ionizing radiation [36,37,38]. There are previous studies which extended to nonionizing radiation where the low (adaptive) dose of ionizing radiation was substituted with RFs [39,40,41,42], intermediate-frequency radiation [43], or UV exposure [44,45].

However, it is necessary to note that the abovementioned studies were conducted mostly with GSM 900/1800 MHz and the effect was sought only with a radiofrequency signal without interaction with other environmental risk factors such as UV. Therefore, research into the effect of combined UV and RF exposure is very important. On the one hand, this corresponds well to our real-life exposure situation; on the other hand, the studied frequency like Wi-Fi (2.4 GHz) is beyond the high frequency range of the third-, fourth-generation mobile phones. It is known that the higher the frequency, the lower the penetration of RF radiation and the more concentrated energy in the first layers of the skin and superficial tissues. Based on this premise, a study of combined exposure to UV and Wi-Fi is more than needed.

The aim of this research was to observe whether two physical agents—both classified by the IARC—in combination have any effect on the human body. Here, we examined whether an RF-EMF (Group 2B) combined with UV radiation (Group 1) has any further outcome on the skin. We used a full-thickness 3D reconstructed skin model to investigate the inflammation and photoaging of the human skin in vitro. The first objective was to investigate the consecutive exposure to a 2422 MHz Wi-Fi RF field and UV radiation (additive effect protocol). The second objective was to investigate the possible adaptive response generated by pre-exposure of the skin to Wi-Fi (as an AD) and subsequent exposure to UV radiation (as a CD) (adaptive response protocol).

## 2. Results

### 2.1. Tissue Viability

MTT results did not show significant changes in the viability percentage (Table 1) in the case of the additive effect protocol.

Under the adaptive response protocol, the MTT results showed a significantly decreased viability percentage value in the RF + UV (*p* = 0.02) and the UV-only treatments (*p* = 0.01) as compared to the sham-exposed treatment (Table 2.)

### 2.2. Interleukins and MMP-1 Enzyme Production

**Figure 1:**
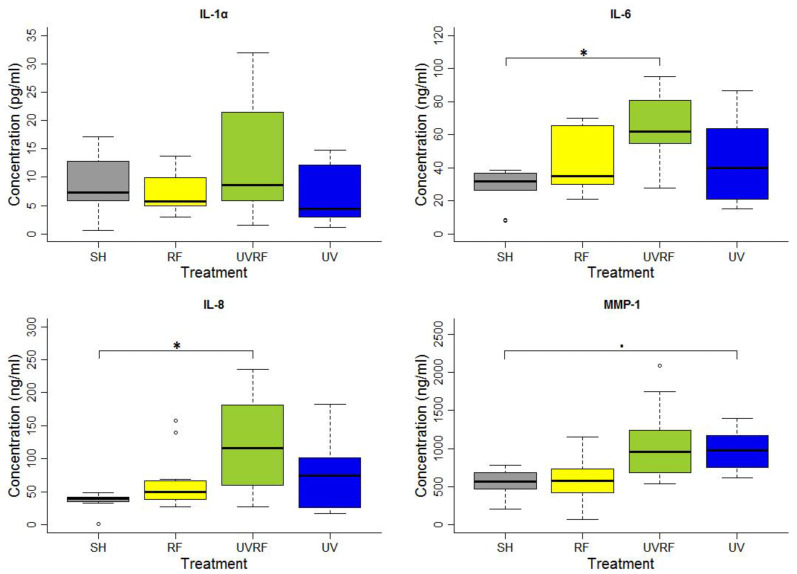
Results of the experiments performed under the additive effect protocol where the skin tissues were exposed first to 2 SED UV and then to 24 h 2422 MHz intermittent 20 min on/off Wi-Fi exposure with 4 W/kg SAR. The data represent three independent experiments. The boxplot shows the median (thick line) and the lower and upper quartiles (the bottom and top lines of the box), and the whiskers show the minimum and maximum values; • = 0.05, * *p* < 0.05, ◦: outlier.

In the experiments performed following the additive effect protocol, the skin tissues were first exposed to 2 SED UV and then to 4 W/kg RF. There were no significant differences in the IL-1α cytokine concentration between the groups of treatments. A significant difference in IL-6 concentration was found between the SH- and UVRF-exposed treatments. There was a significantly increased IL-8 concentration in the UVRF-exposed samples compared to the SH ones. The difference in the concentration of the MMP-1 enzyme was marginally significant between the samples with sham and UV-only exposure. The samples treated with RF radiation were not significantly different from any other samples. Moreover, the UVRF treatment slightly (but not significantly) elevated the IL-1α, IL-6 and IL-8 concentrations as compared to the UV treatment (Figure 1).

**Figure 2:**
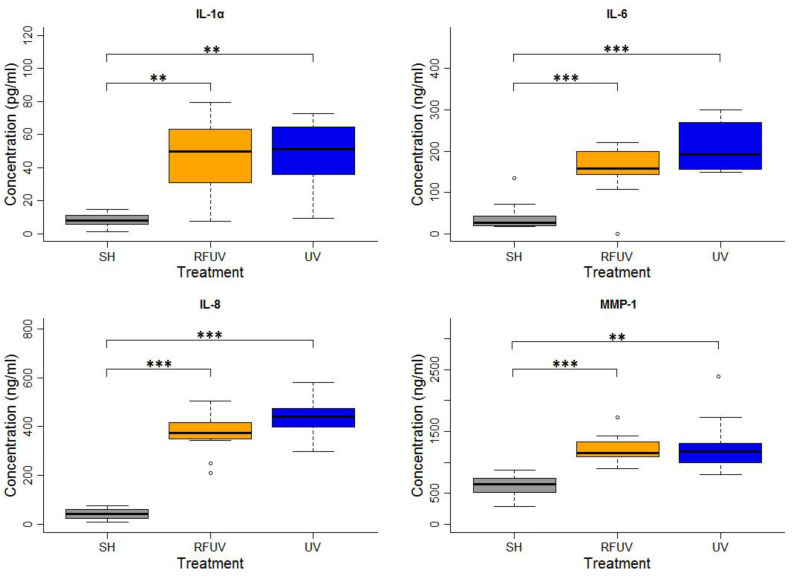
Results of the experiments performed under the adaptive response protocol where pre-exposure of tissues with 2422 MHz continuous Wi-Fi (1.5 W/kg, 24 h) exposure and subsequently with 4 SED UV radiation was performed. The data represent three independent experiments. The boxplot shows the median (thick line) and the lower and upper quartiles (the bottom and top lines of the box), and the whiskers show the minimum and maximum values; ** *p* < 0.005, *** *p* < 0.001, ◦: outlier.

In the experiments performed under the adaptive response protocol, the skin tissues were first exposed to 1.5 W/kg RF and then to 4 SED UV. In these experiments, the concentrations of all types of interleukins, as well as of MMP-1, increased statistically significantly following exposure to either 4 SED UV only or RFUV. Compared to the UV-only exposure, the change of the concentration of all the interleukins and MMP-1 was not significant when the tissues were pre-exposed to RF with 1.5 W/kg SAR for 24 h and subsequently to 4 SED UV radiation (UVRF). Nevertheless, a slight—but not significant—tendency of the protective effects, namely the adaptive response, could be observed in IL-6 and IL-8 production (Figure 2).

## 3. Discussion

While cell phones localize the highest microwave exposure to the brain, Wi-Fi exposure is often localized to other organs, such as the abdomen, leg, and chest area. Some people sleep in rooms with Wi-Fi routers, Wi-Fi baby monitors, or Wi-Fi gaming devices near their pillow. Wi-Fi printers and several wireless devices using the Wi-Fi RF band may be in offices next to a person’s desk and transmit continuously, therefore, the Wi-Fi exposure is quite significant to the overall cumulative exposure within the RF radiation spectrum. Wi-Fi devices emit continuous radiation bursts, just like cell phones, since they always stay in contact with their Wi-Fi router or base station. We spend most of our day indoors while our cell phones connect to the Wi-Fi system immediately. Since modern telecommunications makes it preferable to make a voice call over the Internet, the daily exposure to Wi-Fi has increased compared to mobile communication technologies (2G, 3G, 4G). In this paper, we presented a Wi-Fi exposure of the highest international limit (4 W/kg SAR for the limbs) and an average exposure which could occur indoors (1.5 W/kg SAR).

In a full-thickness (FT) 3D in vitro skin system, normal human-derived epidermal keratinocytes and normal human-derived dermal fibroblasts form a multilayered model of the human dermis and epidermis. This 3D model consists of organized basal, spinous, granular, and cornified epidermal layers analogous to those found in vivo. The dermal compartment is composed of a collagen matrix containing viable normal human dermal fibroblasts. Due to its well-developed membrane structure, 3D in vitro skin systems are realistic models for the examination of skin processes [46,47].

Inflammation is a natural and fundamental response of the body against environmental stimuli and impacts, such as injury, chemicals, pathogens, or radiation. It is a complex process involving biochemical and molecular factors [48]. Inflammation includes the release of growth factors, proinflammatory cytokines, infiltration of inflammatory cells, and ROS production [22]. The interleukin 1 family is a group of cytokines with pro- and anti-inflammatory functions that plays a role in the inflammatory process. Il-1α possesses a strong proinflammatory effect, therefore, it is a key inducer of the inflammatory cascade. It induces keratinocyte secretion of other cytokines, such as TNF-α, IL-6, and IL-8 [49,50]. According to Chung et al., UV radiation might trigger a cutaneous inflammatory response by directly inducing epidermal keratinocytes to elaborate specific cytokines, such as IL-1 and IL-6. They demonstrated that UV-B irradiation upregulates the IL-1α mRNA expression at a lower dose and then downregulates it at a higher dose [29]. Here, we could also detect a significantly increased Il-1, IL-6, and IL-8 production after a 4 SED UV radiation, which is consistent with the findings of other studies [51,52].

The number of studies investigating the effects of inflammation exposed to electromagnetic fields is limited. Extremely low-frequency electromagnetic fields (ELF-EMF) have been reported to be protective against multiple diseases and used in clinical applications. ELF-EMFs modulate chemokine production and keratinocyte growth through inhibition of the NF-κB signaling pathway and reduce IL-8 gene expression [53]. Pulsed ELF-EMFs inhibit the expression of IL-1β [34,54]. On the other hand, Selmaoui et al. observed no effects on interleukin IL-1β and IL-2, but showed a significantly increased IL-6 level as a result of a 50 MHz magnetic field exposure [55].

The results of our present studies suggest that RF exposure alone does not induce inflammatory processes in a 3D skin in vitro model, as found by others [56]. Based on our previous study on the assessment of inflammation exposed to UMTS 1950 MHz RF radiation [40], we decided to investigate whether any of these changes occur as a result of Wi-Fi exposure. Similarly to our previous findings, here, we demonstrate that the cytokines and matrix metalloproteinase-1 production did not change after a 24 h 2422 MHz Wi-Fi exposure.

According to Prasad et al., the expression level of IL-6 is related to the expression of matrix metalloproteinase-1 [57]. Matrix metalloproteinases (MMPs) are zinc-containing endopeptidases with an extensive range of substrate specificities. These enzymes can degrade various components of extracellular matrix proteins, such as collagen, fibronectin, elastin, proteoglycans, etc. MMPs play a role in photocarcinogenesis by regulating various processes related to tumor progression [58]. According to some other researchers, cutaneous exposure to UV irradiation causes the upregulation of several different MMPs. Similarly, we found an increased production of the MMP-1 enzyme due to UV exposure [32,33].

Cela et al. studied the adaptive immune response after repetitive low doses and a single high dose of UV radiation. They demonstrated that the repetitive low doses of UV radiation did not promote an inflammatory state. On the contrary, exposure to a single high UV dose does. Furthermore, they observed a marked suppression of T cell and B cell responses after the single high-dose exposure and an increase in both specific responses after exposure to repetitive low doses [59].

The adaptive response is a well-studied phenomenon. There are several studies which discuss this process, not just in the case of ionizing radiation itself, but also when electromagnetic radiation was challenged by ionizing radiation [39,42,60]. The very first study, which used nonionizing radiation as an adaptive dose, was conducted by Sannino et al. [61]. Freshly collected human peripheral blood lymphocytes were exposed to 900 MHz RF (GSM) radiation and later challenged by mitomycin-C (MMC) or X-ray radiation. These observations suggested that the RF-exposed cells were able to resist the damage induced by subsequent exposure to MMC and thus exhibited an AR. An RF-EMF challenged by UV radiation can also indicate an adaptive response [44,62].

Further studies also indicated that an AR was not elicited instantaneously but required a certain time interval between the AD and the CD to become fully active. Based on this, we set up a protocol which contains a 4 h interval between the AD and the CD. Under the adaptive response protocol, we detected a slight tendency (but not significant) of the adaptive response in cytokine and MMP production when the cells were pre-exposed to 2422 MHz Wi-Fi radiation, later challenged with 1 SED UV radiation.

With the continuously increasing technological development, the issue of radiation exposure emitted by wireless devices has become a subject of research. As 5G and 6G technologies will become omnipresent in modern society, they will bring forward the use of radiation of higher radiofrequencies. The upcoming technologies will use millimeter-range RF-EMFs. As frequency increases, the penetration depth into the human tissue decreases. Thereby, in the millimeter wavelength range, the radiation is absorbed only in the skin of the body [1]. Investigations on the possible health impact of higher frequencies will become necessary and should be observed. According to this, it is necessary to carry out comparative studies such as this paper.

## 4. Materials and Methods

### 4.1. Cell Culture Condition and Protocols

Human skin tissues, which consist of normal human-derived epidermal keratinocytes and normal human-derived dermal fibroblasts, reconstructed with EpiDerm Full Thickness (EFT-300, MaTek Corp, Ashland, MA, USA) were used in these experiments. EFT-300 full-thickness in vitro hydrocortisone-free-reconstructed human skin tissues and a hydrocortisone-free maintenance medium (EFT-300-ASY-HCF) were also purchased from MatTek Corporation and handled according to the manufacturer’s standard user protocols. The tissues were shipped as single-well tissue culture plate inserts at 4 °C on medium-supplemented agarose gel. After arrival, the inserts were transferred into six-well plates containing 1 mL medium, equilibrated at 37 °C, 5% CO_2_ overnight, and maintained in culture until the start of the experiments. EpiDerm FT was maintained in 35 mm Petri dishes filled with 1 mL culture medium throughout the experiments. Two tissues were prepared in parallel for each condition.

### 4.2. Radiofrequency Exposure System

For the Wi-Fi RF exposure, a wire patch cell (WPC) system was used. It is a structure first proposed by Laval et al. [63] operating at 900 MHz; later, it was modified by Paffi et al. [64] to operate at Wi-Fi 2.4 GHz. Briefly, a WPC is constituted by two square parallel metallic plates short-circuited by special props at the corners. The structure is fed through a coaxial cable whose central pin and outer conductor are connected to the ground and the roof, respectively. Four Petri dishes (diameter, 35 mm, and height, 12 mm) can be placed in the four symmetrical regions where the H field presents a quite high and uniform value to take advantage of the inductive coupling. To obtain an easy and repeatable positioning of the biological samples, there is a dielectric round plate (150 mm radius) capable of rotating around the central pin of the feeding cable. The dimensions of the plates (150 × 150 mm^2^), the distance between the plates (15 mm), the radius of the props (5–10 mm) and their distance from the patch edge (8–10 mm) were optimized to set the operating frequency of the WPC in the Wi-Fi frequency band [64]. The temperature control in the biological samples is maintained by using two plate water jackets placed on the external faces of the WPC and connected to a thermostatic bath. The reduced size of the WPC allows it to fit inside an incubator to control the environmental conditions needed for in vitro experiments. To avoid disturbances to the electronic instrumentation of the incubator, the WPC is put inside a metal grid shielding cage (40 × 40 × 20 cm^3^) equipped with six blocks of RF-absorbing material (20 dB of attenuation), one for each side of the cage, to avoid field reflection off metallic walls. Paffi et al. [64] presented a dosimetric study to evaluate the absorbed power (SAR) efficiency ((W/kg)/W) simulated in the whole volume or extrapolated to a thin monolayer on the bottom of a 35 mm Petri dish filled with 1 mL, 2 mL, 3 mL of the culture medium at 24,250 MHz. For the experiments presented in this paper, a new dosimetric study was performed due to the differences in the adopted biological samples.

**Figure 3:**
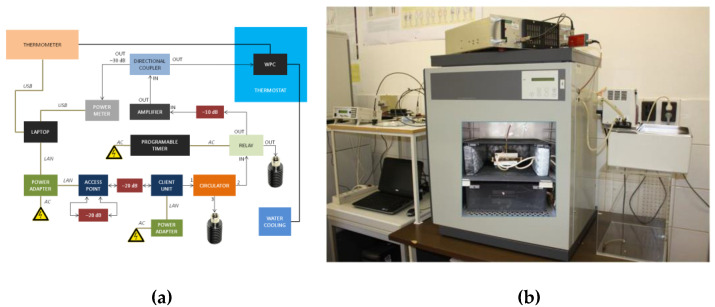
(**a**) Block diagram of a Wi-Fi exposure system; (**b**) arrangement of a whole-exposure system where the wire patch cell (WPC) exposure chambers (see the inserted photograph on the door of the thermostat) were placed in a CO_2_ incubator.

In this experiment, each 35 mm Petri dish filled with 1 mL of the culture medium had a concentric 12 mm inner dish where the skin sample (thickness of 140 µm and diameter of 10 mm) was placed. Simulations were performed using commercial software CST Microwave Studio^®^, 2016. The four dishes were modelled as concentric cylinders of Perspex (ε = 2.6 and σ = 0 S/m) with the wall thickness of 1 mm. The outer dishes were filled with 1 mL of the standard cell culture medium (ε = 77, σ = 2.2 S/m, and ρ = 1000 kg/m^3^). For the skin sample, dielectric properties from the CST library were used. A hexahedral mesh was adopted with 20 lines per wavelength, being the best compromise between accuracy and computational cost. Inside the culture medium and the skin sample, a finer mesh was used (maximum cell dimensions of 0.2 mm and 0.07 mm in all directions, respectively). Open boundary conditions were used to terminate the calculation domain. The SAR distribution within the skin sample was calculated through the option “calculate point SAR” of CST Microwave Studio, 2016. The calculated data on the SAR in the skin sample were extracted and processed to obtain the mean power efficiency and the coefficient of variation (CV) defined as the standard deviation over the mean value that is considered a measure of the SAR inhomogeneity. The simulated mean power efficiency in the skin sample was 0.46 (W/kg)/W, and the CV was 17.3%, respectively. The inhomogeneity was compatible with the value considered acceptable for in vitro experiments [65]. The Wi-Fi signal was generated by an access point (AP) and a client unit (CU) operated at 2422 MHz frequency. The AP and the CU units were both controlled with software under MS Windows. To achieve the on/off RF exposure, a timer and an RF switch relay were used [66]. The Wi-Fi signal generated by AP/CU units was amplified by a solid-state amplifier (Milmega, Ryde, UK) up to the requested power level (Figure 3). The Wi-Fi signal was set (IEEE 802.11 b/g) with the uploading speed of 30 Mb/s. The characteristics of modulation of the Wi-Fi signal were measured and the temperature in the sample was controlled during the exposure.

The SAR of the exposed 3D tissues depended on the study performed by combined exposure under the additive effect protocol or the adaptive response protocol. In the case of the additive effect protocol, the samples were exposed to Wi-Fi with 20 min on/off switch timeframes during the 24 h exposure period where the SAR was 4 W/kg. Under the adaptive response protocol where the possible adaptive response was investigated, 24 h of continuous exposure to Wi-Fi with 1.5 W/kg of the SAR (as an adaptive dose) were used.

### 4.3. Ultraviolet Exposure System

**Figure 4:**
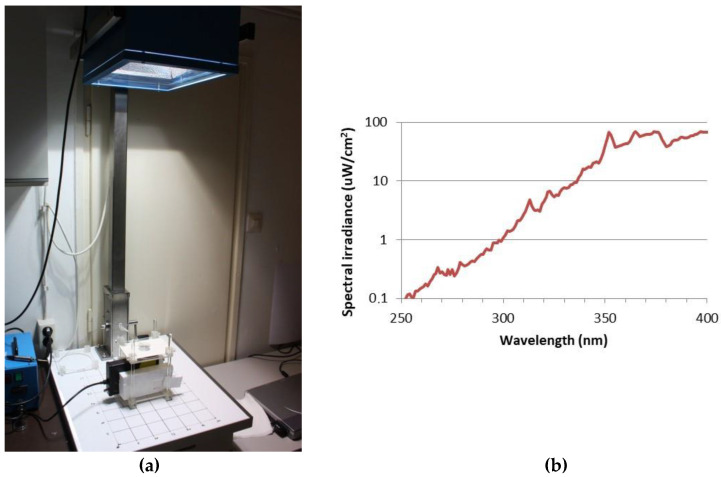
(**a**) Spectral measurement, the UV exposure system of the solar simulator (Hönle SOL-500) equipped with a wavelength filter; (**b**) spectral irradiance of the solar simulator filtered with the lid of a Petri dish.

The UV exposure system was described earlier [40]. Skin tissues were transferred to 35 mm Petri dishes, filled with 1 mL Dulbecco’s phosphate-buffered saline (DPBS) and exposed to 2 SED or 4 SED UV radiation. For irradiation, a solar simulator SOL-500 (Hönle UV Technology, Gilching, Germany) was used, which was equipped with an H2 filter that transmits wavelengths ≥ 295 nm (UVB, UVA, visible light). The lamp was set at a distance of 49 cm, with which the tissues were exposed for 30 or 60 min depending on the dose (2 or 4 SED) (Figure 4a). The dose was verified with a calibrated spectroradiometer (ILT-900, International Light Technologies, Peabody, MA, USA) before exposure. Because plastic materials of cell culture holders contain UV stabilizers, the transmitted spectrum was measured through the same type of a 35 mm Petri dish lid that was used in the assay (Figure 4b). According to the results of preliminary experiments on the viability of skin tissues and cytokine production, in the additive effect protocol, the applied UV dose was 2 SED, while in the adaptive response protocol, the UV dose was set to 4 SED. During the UV exposure, the samples in the Petri dishes were placed on a metal heat exchanger that was connected to a thermal bath with tubes so the temperature-controlled water could circulate. The temperature of the water in the bath was set in a way to keep the constant temperature below 37 °C in the samples. The temperature of DPBS in the Petri dishes was monitored throughout the whole duration of the UV exposure, and it was kept at 35.5–37.0 °C.

### 4.4. Experimental Protocols

The sequence of exposures to UV and RFs depended on the appropriate study protocol.

**Figure 5:**
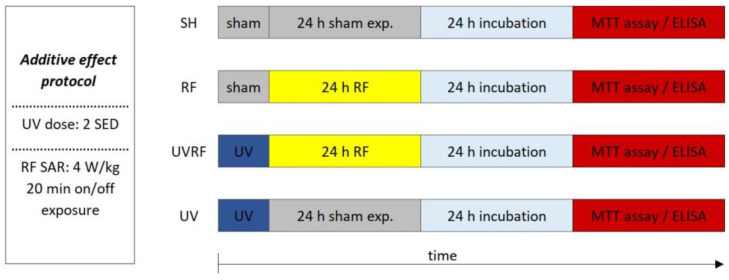
Timeline of the additive effect protocol where 2 SED UV irradiation was followed immediately by 24 h RF irradiation with 4 W/kg.

In the case of the first study—for better understanding called the additive effect protocol—2 SED UV irradiation (as the first exposure) was followed immediately by 24 h of RF irradiation with 4 W/kg as the second exposure (Figure 5).

The following four exposure conditions were examined in the additive effect protocol:

- sham exposure (SH),
- 4 W/kg RF exposure (RF),
- 2 SED UV exposure followed by 4 W/kg RF exposure (UVRF),
- 2 SED UV exposure (UV).

**Figure 6:**
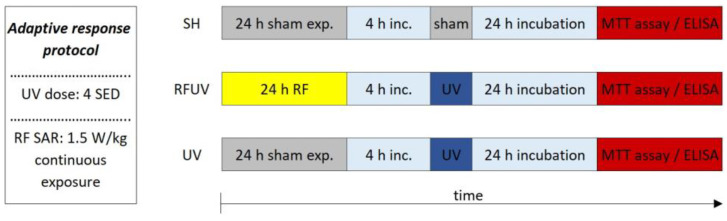
Timeline of the adaptive response protocol.

Under the second study—called the adaptive response protocol—the tissues were pre-exposed to RFs (24 h, 1.5 W/kg) and, after a 4 h incubation period, 4 SED UV radiation (Figure 6) was applied.

The following three exposure conditions were examined in the adaptive response protocol:

- sham exposure (SH),
- 1.5 W/kg RF exposure followed by 4 SED UV exposure (RFUV),
- 4 SED UV exposure (UV).

Different UV doses were chosen because in case of the possible cooperative effect (additive effect protocol), a lower UV dose (2 SED) is needed to detect if there might be additional effects from RF exposure, while in the case of the adaptive response protocol the protective effect is more detectable if the effect of exposure to UV is stronger due to a higher (as a challenge 4 SED) UV dose.

In these procedures, 24 h incubation was necessary after the protocols to evaluate inflammation and photoaging so the secretions of MMP-1 and interleukin concentrations could reach the highest values. Three independent experiments were carried out.

### 4.5. Assay Procedures

#### 4.5.1. Cell Viability Analysis (MTT Assay)

Cell viability analysis was evaluated using the 3-(4,5-dimethylthiazol-2-yl)-2,5-diphenyltetrazolium bromide (MTT) method with an MTT kit (MTT-300, MatTek, Ashland, MA, USA). The inserts were transferred to the cell viability assay immediately after the experimental protocol (exposure followed by 24 h incubation). The EFT-300 tissue samples were placed in a 24-well plate containing 300 µL/well of the MTT solution and incubated at 37 °C, 5% CO_2_ for 3 h. Afterwards, the tissues were transferred into a new 24-well plate and immersed in the 2 mL/well extraction solution (isopropanol) and incubated for 2 h at room temperature on a shaker, in the dark. After the extraction process, the solutions in each well were suspended and 200 µL/sample were transferred into a 96-well plate. Absorbance at the 570 nm and 650 nm wavelengths were measured as the test and background wavelengths using a microplate reader (Anthos 2010, Biochrom, Cambridge, UK). The percentage of viability in the MTT test was determined for each tissue using the following equation: viability = 100 × OD_(sample)_ / OD_(negative control)_. The percentages above 50% were considered to indicate viable cells according to the tissue manufacturer’s instruction, which follows OECD Guideline No. 439 [46,67].

#### 4.5.2. Inflammation and Photoaging

At the end of exposure, after the 24 h incubation period, the culture media from the Petri dishes were collected and stored at −20 °C until measurements. The MMP-1 enzyme and cytokines (IL-1α, IL-6, IL-8) were measured in the culture medium using ELISA kits (Thermo Fisher Scientific, Waltham, MA, USA) according to the manufacturer’s instructions.

### 4.6. Statistical Analysis

In each of the three independent experiments, there were two biological parallels for each treatment type. For the MTT assay, the mean percentage of viability was calculated for each treatment. Data from the three independent experiments were statistically analyzed using ANOVA. Data on the concentration of interleukins and MMP-1 enzymes were analyzed using linear mixed-effect models with Tukey’s post-hoc test. The significance level was set at *p* < 0.05. All the analyses were performed using RStudio software version 1.1.463.

## 5. Conclusions

This study aimed to investigate whether Wi-Fi exposure combined with UV radiation has any effect on the inflammation process in the skin. Here, we exposed 3D reconstructed human skin tissues to UV and 2422 MHz RF consecutive exposure—in different sequences—to investigate the inflammatory process of the skin in vitro. In both sequences, we noticed a slight tendency for 2422 MHz Wi-Fi exposure-associated changes in the factors which have an important role in inflammation. Overall, we could not detect a significant adaptive response (protective effects) by Wi-Fi irradiation against ionizing radiation. As we develop telecommunication techniques involving higher frequencies of RF-EMFs, the radiation is absorbed mainly in the skin. With the introduction of the 5G and 6G technologies, further investigations on the skin will become fundamentally crucial.

## Figures and Tables

**Table 1:** Cell viability results under the additive effect protocol with 2 SED UV exposure followed by 24 h of 20 min on/off switch 4 W/kg SAR Wi-Fi exposure.

Additive Effect Protocol		
Treatment	Viability Percentage	
	Mean ± SD	*p*-value ^1^
SH	100 ± 0.0	
RF	99.15 ± 3.17	0.99
2 SED UV + RF	91.95 ± 3.89	0.24
2 SED UV	89.54 ± 8.16	0.1

^1^ Note: *p*-values represent the comparison to the SH treatment.

**Table 2:** Cell viability results under the adaptive response protocol with the 24 h continuous 1.5 W/kg SAR Wi-Fi exposure followed by 4 SED UV exposure.

Adaptive Response Protocol		
Treatment	Viability Percentage	
	Mean ± SD	*p*-Value ^1^
SH	100 ± 0.0	
RF + 4 SED UV	72.93 ± 12.23	0.018 *
4 SED UV	68.73 ± 8.15	0.009 **

^1^ Note: *p*-values represent the comparison to the SH treatment (* *p* < 0.05. ** *p* < 0.005).

## Data Availability

Not applicable.
